# The Efficacy of Probiotics in the Management of Helicobacter Pylori: A Systematic Review

**DOI:** 10.7759/cureus.20483

**Published:** 2021-12-17

**Authors:** Sai Sri Penumetcha, Saher Ahluwalia, Rejja Irfan, Sawleha Arshi Khan, Sai Rohit Reddy, Maria Elisa Vasquez Lopez, Maryam Zahid, Alberto Busmail, Lubna Mohammed

**Affiliations:** 1 Internal Medicine, California Institute of Behavioral Neurosciences & Psychology, Fairfield, USA; 2 Internal Medicine, Brooklyn Medical Services, New York, USA; 3 Research, California Institute of Behavioral Neurosciences & Psychology, Fairfield, USA; 4 Gastroenterology and Hepatology, Mayo Clinic, Rochester, USA; 5 School of Medicine, Armed Forces Medical College, Pune, IND; 6 Research and Development, California Institute of Behavioral Neurosciences & Psychology, Fairfield, USA

**Keywords:** hpylori, probiotics and microbiome, gastritis, eradication, dietary supplements, yogurt

## Abstract

*Helicobacter pylori* is a Gram-negative microorganism that causes chronic dyspepsia, gastritis, mucosa-associated lymphoid tissue (MALT) lymphoma, and gastric adenocarcinoma. Various antibiotic regimens are employed to eradicate it; however, antibiotic resistance has skyrocketed in recent years, resulting in a reduction in eradication rates. As a result, numerous novel therapeutic approaches are being adopted in clinical practice, and probiotics are being extensively investigated. Probiotics are living bacteria that, when consumed, offer many medicinal advantages that may be accomplished by altering the amount or activity of gut flora. Their beneficial influence on gut health, immune system modulation, and cancer therapy is the subject of extensive investigation. This is owing to their perceived safety and simplicity of use. The primary objective of this review is to learn about and investigate the function of probiotics in the eradication of *H. pylori*, either alone or in conjunction with traditional treatments.

Data have been collected from PubMed, PubMed Central, Medline, Cochrane, and Google Scholar, and relevant articles have been chosen following the PRISMA guidelines. Our search resulted in 2489 records, of which 123 full-text articles were screened for eligibility. Two reviewers independently performed the quality appraisal of 16 relevant articles, and ultimately 11 high-quality studies are included in this review. In conclusion, probiotic monotherapy does not have a significant effect on the eradication rates of *H. pylori*, but in conjunction with standard treatment regimens, there was mild improvement in the eradication rates but a significant reduction of side effects due to antibiotics.

## Introduction and background

Infection with *Helicobacter pylori* is one of the most prevalent chronic bacterial illnesses in humans, affecting roughly 4.4 billion people globally [1]. *H. pylori* is a Gram-negative, flagellated bacterium that inhabits the human stomach and causes prolonged gastric inflammation leading to gastritis, predisposing to gastric cancer and MALT lymphoma [1,2]. Gastric cancer constitutes nine percent of all cancer-related deaths, and hence *H. pylori* eradication has been demonstrated to lower the incidence of stomach cancer [1]. Until recently, triple therapy consisting of omeprazole, amoxicillin, and clarithromycin was considered the conventional first-line regimen. However, antibiotic resistance caused by the emergence of point mutations leads to increased failure rates [2]. In developed nations like Europe and the United States of America, quadruple therapy, which includes bismuth sub-citrate potassium, metronidazole, tetracycline, and omeprazole, is suggested as the primary treatment [3]. The quadruple regimen continues to have significant adverse effects like abdominal pain, nausea, vomiting, and bloating, leading to poor patient adherence to the medication, which further encouraged the emergence of antibiotic-resistant strains, and therefore, new treatment options are being explored [4].

Probiotics are live microorganisms, which when ingested, have numerous medical benefits that can be achieved by modifying the quantity or activity of the gut flora [5]. The two main species utilized as probiotics are *Lactobacillus* and *Bifidobacterium*, which are non-spore-forming, Gram-positive rods [6]. The resilience to low pH and endurance to a broad variety of temperatures define these bacteria, and their native habitat is the digestive system, oral mucous membranes, and genital tracts. These are being widely researched for their positive impact on gut health, immune system modification, and cancer therapies [6]. These dietary supplements are gaining popularity due to their perceived safety and ease of usage [5].

The selected suitable antimicrobials, their optimal combination, dosage, frequency, duration of treatment, and the necessity to use adjuvants are the parts of successful management of an infectious process [7]. Any disturbance in the equilibrium of the beneficial and harmful microorganisms in the body can trigger an inflammatory response, which causes epithelial malfunction [8]. *H. pylori* alter the host microbiome, most likely through changes in the gut microenvironment and acid-base balance. Hence probiotic supplementation may be beneficial in the treatment of inflammatory and infectious diseases [8,9]. Some studies have shown that adding probiotics to the traditional antibiotic regimen in *H. pylori* treatment can minimize the adverse effects of the antibiotics, most likely by re-establishing the balance of intestinal microbes [3]. Also, some of the probiotics are known to improve the success rates of *H. pylori* eradication, by both immunological and non-immunological mechanisms, like inhibiting* H. pylori *adhesion to the mucous membrane or by altering the pH [9]. However, other investigations have revealed that the use of probiotics has not improved the outcomes [4]. Furthermore, depending on the mechanism of action of various strains, pairing several strains of probiotics may be useful for boosting the eradication rate of infection [10]. These conflicting results have led to clinical confusion as to how this condition is best treated. 

The primary goal of this systematic review is to determine the therapeutic value of probiotics, either alone or in conjunction with conventional treatment regimens, in the management of *H. pylori* eradication in adults.

## Review

Methods

Relevant studies were found by searching PubMed, PubMed Central, Medline, Cochrane Library, and Google Scholar. This review was carried out using the preferred reporting items for systematic review and meta-analysis (PRISMA) guidelines. The following keywords were used for the search: *Helicobacter pylori*, *Campylobacter pylori*, *proteobacteria*, *Helicobacteraceae*, Gram-negative bacteria, *Spiral Bacterium*, eradication, gastritis, *Lactobacillus*, *Bifidobacteria*, *Saccharomyces*, probiotics, yogurt, microbiome, and dietary supplements. Additionally, a combination of the above regular keywords and MeSH strategy was used to identify relevant records from the PubMed databases.

**Figure 1:**
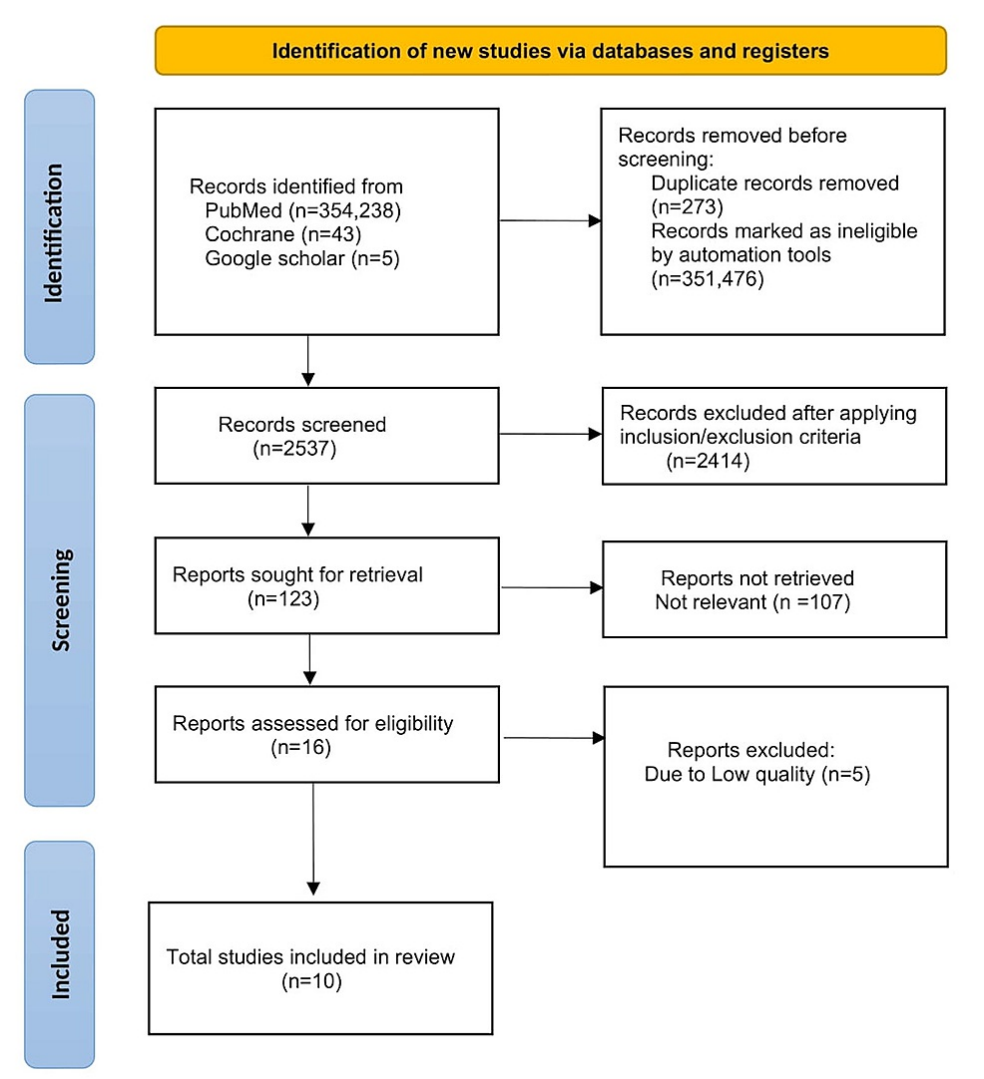
PRISMA Flow Diagram PRISMA: preferred reporting items for systematic reviews and meta-analysis [11]

Only studies that satisfied the following criteria were considered: (1) research conducted in the previous 10 years, which is 2011 through 2021, (2) free full-text articles, (3) studies conducted only on human subjects, (4) patient population 18 years or older. All animal studies, articles in languages other than English, books, and documents were excluded. Furthermore, research and review articles published before 2011 were omitted to provide a greater emphasis on the most recent advancements in therapies while keeping antibiotic resistance in mind. The literature selection process is outlined in the following PRISMA flow diagram [11] (Figure 1).

Results

Our search yielded 354,238 records from PubMed, PubMed Central, and Medline; 43 records from Cochrane, and five records from Google scholar. After eliminating duplicates and irrelevant articles, 123 full-text articles were screened for eligibility. For our data analysis, we used EndNote basic (Clarivate, London, United Kingdom) as a reference manager. Sixteen studies were pre-qualified for quality evaluation out of 123. Two authors independently analyzed the results of these 16 papers using the standardized tools listed under; the Cochrane quality assessment tool for randomized clinical trials and the AMSTAR Checklist (Ottawa, Canada) for systematic reviews. This review eventually incorporated 10 studies. Table 1 summarizes the findings of the studies included in this review, as well as the conclusions drawn from each. 

**Table 1:** Study analysis and review *H. pylori*: *Helicobacter pylori*, ITT: intention to treat, RCT: randomized control trial, BQT: bismuth containing quadruple therapy, GI: gastrointestinal

No	Authors	Year Published	Study design	Study Population	H.pylori Diagnostic test	Eradication Regimen	Probiotics used	Outcomes
1	Giuseppe Losurdo [2]	Jan-18	Systematic review	11 studies were selected. Probiotics eradicated *H. pylori* in 50 out of 403 patients.	N/A	Probiotic monotherapy	N/A	Probiotics alone show a minimal effect on *H. pylori* clearance, suggesting a likely direct role.
2	Lynne V McFarland [4]	Oct-15	Systematic review and meta-analysis	19 RCTs with 2730 participants using six different types of multi-strain probiotics	N/A	N/A	N/A	Adjunctive use of some multi-strain probiotics may improve *H. pylori* eradication rates and prevent the development of adverse effects and, but not all mixtures were effective
3	Maria Pina Dore [7]	Apr-19	Randomized control trial	56 *H. pylori* positive subjects assigned in 1:1 ratio into active or placebo group	Upper endoscopy with biopsy and 13C urea breath test	Probiotics and Pantoprazole or Placebo and Pantoprazole	Limosilactobacillus reuteri	The cure rate in the active group is 10.7% for ITT analysis versus 3.5% for the placebo group
4	Jin Young Yoon [8]	Jul-19	Randomized control trial	142 patients allocated to treatment or placebo group	13C Urea Breath test, campylobacter-like organism test or histological examination	Subjects were randomized in 1:1 ratio into fermented milk with *Lactobacillus paracasei* HP7 and *Glycyrrhiza glabra* group or fermented milk only group for eight weeks	*Lactobacillus paracasei* and *Glycyrrhiza glabra*	The combination of *Lactobacillus paracasei* and *Glycyrrhiza glabra* improved GI symptoms, reduced *H. pylori* density and inflammation
5	Luyi Chen [9]	Aug-18	Randomized control trial	70 *H. pylori* positive patients were randomized into two groups	Esophagogastroduodenoscopy and histology	Bismuth containing quadruple therapy versus probiotics, and Bismuth containing quadruple therapy for 14 days	Clostridium butyricum	Eradication rates were 88.6% for the BQT group and 85.7% for probiotic and BQT group. No significant differences in eradication but there was a significant improvement in GI symptoms
6	Rahmatollah Rafiei [10]	Dec-20	Randomized control trial	106 *H. pylori* positive patients were assigned into two groups	Endoscopy	Triple therapy or probiotics and triple therapy	*Lactobacillus rhamnosus*, *Lactobacillus casei*, *Streptococcus thermophiles*, *Lactobacillus bulgaricus*, *Lactobacillus acidophilus*, *Bifidobacterium breve*, and *Bifidobacterium longum*,	The eradication rate is 88.5% in the probiotic and triple therapy group and 63.3% in triple therapy group
7	Wen Ji [11]	Sep-18	Randomized control trial	526 *H. pylori* positive patients were randomized into two groups in equal ratio	14C-Urea breath test and electronic gastroscopy	Quadruple therapy or quadruple therapy with probiotics for 14 days	Compound *Lactobacillus acidophilus*	N/A
8	Ryuzo Deguchi [12]	Nov-11	Randomized control trial	229 patients with *H. pylori* were randomized into two groups	A bacterial culture or rapid urea breath test	Triple therapy or probiotics and triple therapy for one week along with pretreatment with probiotics alone for three weeks	*Lactobacillus gasseri* containing yogurt	The eradication rate for the probiotic group was 38.5% versus 28% for the triple therapy alone group.
9	Jian Zhang [13]	Sep-20	Randomized, single-arm pilot trial	150 positive patients were randomly allocated to different probiotic regimens	C13/14-Urea Breath Test, rapid urease test, stool antigen test or histology exam and culture	*Clostridium butyricum* in group A, *Bacillus coagulans* in group B and C. *butyricum* plus B *coagulans* in group C for eight weeks	*Clostridium butyricum* and *Bacillus coagulans*	The eradication rates were 18%, 20% and 26% in three groups, respectively. Hence C *butyricum* and B *coagulans* may effectively inhibit *H. pylori* to some extent.
10	Muhan Lu [14]	Oct-16	Meta-analysis of randomized control trials	13 randomized controlled trials, including 2306 patients	N/A	N/A	N/A	Probiotic supplementation may be effective in improving eradication rates and alleviating the disease related adverse effects

Discussion

*Helicobacter pylori* have been known to infect humans for about 58,000 years, yet it wasn't found until 1982 [15]. *H. pylori* have been extensively studied by scientists all over the world since its isolation and culture by J. Robin Warren and Barry J. Marshall in 1982 [16]. Potentially treatable conditions caused by *H. pylori* include dyspepsia, peptic ulcer disease, Mucosa-associated lymphoid tissue lymphoma, and gastric adenocarcinoma [15]. It is surprising to learn that *H. pylori* are related to several diseases that are just not gastroenterological but also include several cardiovascular, hematological, and neurological conditions [15]. Probiotics generate bacteriostatic chemicals that limit *H. pylori* colonization and minimize treatment-related adverse effects such as antibiotic-associated diarrhea [16]. They also play an increasingly important role in health and disease, and there is an expanding body of data on the therapeutic potential of probiotics in many gastrointestinal diseases, including irritable bowel syndrome and other conditions [17]. However, several studies have contradictory findings on the efficacy and safety of probiotics in aiding with eradication [16]. Despite earlier research focusing on probiotic addition, the time and duration of probiotic treatment remain unknown.

This systematic review was conducted to investigate in depth and gain a better understanding of what is known, what is unknown, and what needs to be focused on in the future for the management of *H. pylori* and the significance of probiotics in the same. As a result, we have included a brief overview of *H. pylori*’s pathophysiology, its associations, typical treatment regimens, and antibiotic resistance to assist us in going through our review.

H. Pylori Pathogenesis and Its Associations

*H. pylori* infection is extremely common, possibly affecting more than half of the world’s population [18]. Africa has the highest prevalence (79.1%), followed by Latin America and the Caribbean (63.4%) and Asia (54.7%). North America (37.1%) and Oceania (24.4%), on the other hand, have the lowest *H. pylori *prevalence rates [19]. Pathogenic variables linked with chronic *Helicobacter* infection include its motility, bacterial chemotaxis, adherence, and CagA positive strains [20]. Chronic gastritis induced by *H. pylori* infection progresses to gastric adenocarcinoma, which develops as a result of a chain of events that includes gastritis, atrophy, intestinal metaplasia, dysplasia, and cancer [18]. The risk of gastric carcinogenesis in individuals infected with Cag PAI (Cag pathogenicity island) positive strains is double that of Cag PAI-negative strains [20]. Gastric epithelial proliferation and carcinoma development are induced by CagA transgenic expression in mice, and CagA suppresses apoptosis both in vitro and in vivo, indicating that CagA is a bacterial oncoprotein [21].

**Figure 2:**
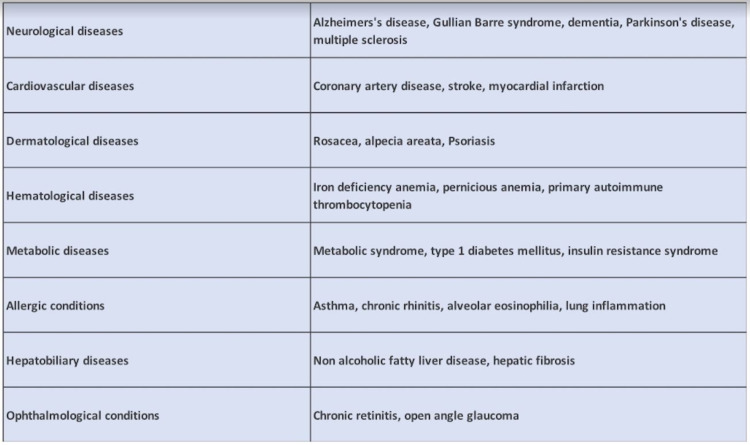
Helicobacter pylori associations

*H. pylori *colonization is known to be related to a variety of diseases. Some of its disease associations include Alzheimer’s disease, multiple sclerosis, rosacea, psoriasis, pernicious anemia, iron deficiency anemia, chorioretinitis, insulin resistance syndrome, Type 1 DM, metabolic syndrome, stroke, asthma, and many more [22]. This bacterium has been linked to an increased incidence of both diffuse and intestinal gastric cancer [18]. As a result of all these clinical interconnections and the most frequent gastric and duodenal ulcers, we must eliminate its colonization [18,22]. Figure 2 demonstrates some of the associations of *H. pylori*.

Management and Antibiotic Resistance

Multiple regimens are available to treat *H. pylori *infection. The most popular regimens are triple therapy which consists of standard proton pump inhibitor, clarithromycin, and amoxicillin, and quadruple therapy with standard proton pump inhibitor, bismuth, tetracycline, and metronidazole [23]. Other less common but emerging regimens are sequential therapy, concomitant therapy, hybrid therapy (sequential-concomitant therapy), quinolone-based therapy, and salvage therapy [23]. The Maastricht V consensus recommended quadruple therapy containing PPI, bismuth, and two antibiotics as the first-line therapy for *H. pylori* eradication [12].

In the early 1990s, triple therapy consisting of a proton pump inhibitor, clarithromycin, and amoxicillin (CAM) was widely used, and the cure rate was about 80% [24]. Eventually, various factors led to the development of antibiotic resistance, which reduced the cure rates and brought the need for new treatment regimens. The most important factors are poor patient compliance, greater bacterial loads, biofilm formation, improper and insufficient regimens, and gene polymorphisms [24]. Prolonged treatment with such high dose antibiotics also caused increased adverse effects like imbalance in the gut microbial environment, antibiotic-related diarrhea, and *Clostridium difficile* infections, which further lead to poor compliance and antibiotic resistance [12]. Another vital factor for the failure of eradication is CAM (clarithromycin and amoxicillin) resistance due to 23S rRNA gene mutation [13].

Role of Probiotics

Researchers believe that probiotics may help treat diarrhea, constipation, irritable bowel syndrome, and other gastrointestinal disorders [14]. Several experimental investigations have suggested that different probiotics may have an antagonistic impact on *H. pylori;* however, the specific mechanisms are yet to be discovered [25]. *Lactobacillus* predominates in the non-acidic areas of the stomach, and *Saccharomyces* predominates in the acidic areas [26]. Chronic *H. pylori* infection reduces the *Lactobacillus* density in the stomach as well as alters the quality and quantity of the gut microbial flora [26]. The capacity of probiotics to inhibit *H. pylori *from attaching epithelial cells is typically caused by several processes such as competing for adhesion sites or nutrients, creating steric hindrance, and secreting antimicrobial compounds [25]. Additionally, probiotics also can partially stabilize or restore normal endogenous microbiota and suppress *H. pylori* growth [26]. Few studies also mentioned that probiotics increase IgA production, which supports the mucosal barrier against pathogens [25]. Hence they have recently been proposed as a novel therapeutic option against *H. pylori* [26]. However, this increase in the *H. pylori*eradication rates and reduction in adverse responses may be strain-specific [27].

In this study, we looked at the impact of several kinds of probiotics on *H. pylori* eradication, either alone or in conjunction with other conventional regimens. In a randomized controlled study with *Limosilactobacillus Reuteri*, Maria Pina Dore et al. reported that probiotics combined with pantoprazole had an eradication rate of 10.7% compared to 3.5% in the pantoprazole alone group [7]. In another study, Luyi Chen et al. found that bismuth quadruple therapy plus *Clostridium* had no significant difference in eradication rates when compared to bismuth quadruple therapy alone [9]. In contrast, Jian Zhang et al. found in their study that *C. butyricum* and *B. coagulans* can eliminate *H. pylori* to some level and can be utilized as an alternate therapy in antibiotic-resistant individuals [14]. When clarithromycin-based triple therapy was combined with probiotics, tolerance rose to more than 85%, and GI symptoms decreased from 76% in the usual triple therapy to 15% [28]. Furthermore, as indicated by Guiseppe Losurdo, Muhan Lu, and Lynne V McFarland's systematic reviews and meta-analysis, probiotics may have a little influence in the eradication of *H. pylori* and aid in the reduction of antibiotic-related adverse effects. These three reviews together looked at a total of 43 randomized control studies [2,4,26].

Concurrent probiotic and eradication therapy administration is usually believed to be more effective than other treatment regimens; nevertheless, clinical investigations have found varying outcomes about the optimal periods for providing probiotics [14]. However, most studies concluded that probiotics alone had no meaningful influence on eradication, although they did minimize the adverse effects of extended antibiotic therapy. This might be attributed to the probiotics repairing the disrupted microbial ecology in the stomach, which helped minimize nausea, GI distress, or diarrhea-like side effects and led to improved patient compliance. Thus, probiotics, when used in conjunction with conventional treatment regimens, showed a substantial improvement in eradication rates, particularly when used in conjunction with quadruple or triple therapy, while probiotic monotherapy had unsatisfactory results.

Limitations

Our study has numerous limitations, including a small number of publications included, evaluation of only free full-text papers, small sample sizes, and no consideration of recurrence rates; hence, any conclusions reached here are based on restricted data. Our findings should be interpreted with caution due to the high level of heterogeneity across the studies included in our analysis. Antibiotic resistance varies by area, and so eradication rates vary accordingly.

## Conclusions

In conclusion, probiotics alone do not play a role in the eradication of *Helicobacter pylori*, but when combined with traditional treatment regimens, there is a slight increase in the eradication rates, which could be due to a reduction in the antibiotic-associated side effects, which leads to better patient adherence to the regimen. Furthermore, their effect might be strain-specific, dose-specific, or duration-specific. Additionally, pretreatment with probiotics, as well as supplementation throughout the therapy, resulted in higher eradication rates. The current comprehensive analysis showed significant evidence for the therapeutic benefit of probiotics in decreasing antibiotic-associated adverse effects. Though *H. pylori* eradication is studied worldwide, it has mostly been investigated in certain geographical locations such as China, Korea, Europe, and the Middle East. Hence, more well-designed, randomized, and thorough studies with high sample sizes in diverse locations should be undertaken, taking into account antibiotic resistance dependent on geography. It is also necessary to investigate the precise mechanism by which probiotics affect *helicobacter* elimination. As previously stated, only a few probiotics have a favorable impact; hence future researchers must investigate the precise strain and dose, as well as the specific duration, to improve the outcome of this chronic illness.
